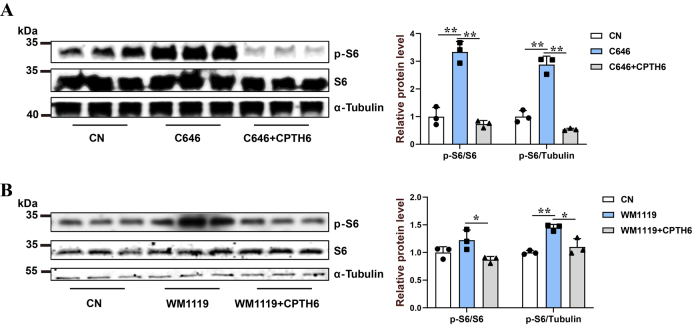# Inhibition of mitochondrial fatty acid β-oxidation activates mTORC1 pathway and protein synthesis *via* Gcn5-dependent acetylation of Raptor in zebrafish

**DOI:** 10.1016/j.jbc.2023.105220

**Published:** 2023-09-03

**Authors:** Wen-Hao Zhou, Yuan Luo, Rui-Xin Li, Pascal Degrace, Tony Jourdan, Fang Qiao, Li-Qiao Chen, Mei-Ling Zhang, Zhen-Yu Du

**Affiliations:** 1 LANEH, School of Life Sciences, East China Normal University, Shanghai, P.R. China; 2 Pathophysiology of Dyslipidemia Research Group, INSERM UMR1231 CTM (Center for Translational and Molecular Medicine) Ex-Lipids, Nutrition, Cancer, Université de Bourgogne Franche-Comté, Dijon, France

**Keywords:** mitochondrial FAO inhibition, mTORC1, acetyl-CoA, raptor, Gcn5

## Abstract

Pharmacological inhibition of mitochondrial fatty acid oxidation (FAO) has been clinically used to alleviate certain metabolic diseases by remodeling cellular metabolism. However, mitochondrial FAO inhibition also leads to mechanistic target of rapamycin complex 1 (mTORC1) activation–related protein synthesis and tissue hypertrophy, but the mechanism remains unclear. Here, by using a mitochondrial FAO inhibitor (mildronate or etomoxir) or knocking out carnitine palmitoyltransferase-1, we revealed that mitochondrial FAO inhibition activated the mTORC1 pathway through general control nondepressible 5–dependent Raptor acetylation. Mitochondrial FAO inhibition significantly promoted glucose catabolism and increased intracellular acetyl-CoA levels. In response to the increased intracellular acetyl-CoA, acetyltransferase general control nondepressible 5 activated mTORC1 by catalyzing Raptor acetylation through direct interaction. Further investigation also screened Raptor deacetylase histone deacetylase class II and identified histone deacetylase 7 as a potential regulator of Raptor. These results provide a possible mechanistic explanation for the mTORC1 activation after mitochondrial FAO inhibition and also bring light to reveal the roles of nutrient metabolic remodeling in regulating protein acetylation by affecting acetyl-CoA production.

Mitochondrial fatty acid (FA) oxidation (FAO) involves a series of reactions in which FAs are progressively broken down to produce acetyl-CoA that mainly enter the tricarboxylic acid cycle to generate energy (ATP or ketone bodies, depending on the tissue) (1). Mitochondrial FAO alteration linked to the deficiency of specific FA transport proteins or β-oxidation is associated with the development of various pathologies, such as liver dysfunction, hypoglycemia, Reye-like syndrome, skeletal myopathy, cardiomyopathy, and arrhythmias (2, 3, 4, 5). Mitochondrial FAO inhibition has been reported to induce metabolic remodeling and thus could be used to improve glucose homeostasis *in vivo* by promoting glucose metabolic utilization (6, 7, 8, 9, 10, 11). In this context, mildronate (MD), a drug that inhibits mitochondrial FAO through competitive inhibition of l-carnitine synthesis, has been shown to promote glucose uptake and oxidative catabolic utilization, thereby improving ischemic heart disease and insulin resistance (12, 13, 14).

The inhibition of mitochondrial FAO could promote amino acid (AA) and protein synthesis, and cause cell proliferation and/or tissue hypertrophy, either in the clinic or in the experiment on human and animal (15, 16). Studies have shown that tissue hypertrophy induced by mitochondrial FAO inhibition is highly correlated with mechanistic target of rapamycin complex 1 (mTORC1) pathway activation (17) and its downstream effector ATF4, which promotes AA synthesis and transport (16, 18, 19, 20, 21, 22, 23, 24). In previous studies performed in fish, we have also found that inhibition of mitochondrial FAO by MD or carnitine palmitoyltransferase-1 (Cpt-1) knockout activates the mTORC1 signaling pathway that was associated to an increase in the size and mass of liver and muscle tissue and in protein deposition and growth performance (25, 26). Therefore, mitochondrial FAO inhibition–induced mTORC1 pathway activation and concomitant cell proliferation/tissue hypertrophy effects are conserved among species with universal biological significance. However, the exact mechanisms underlying the mTORC1 pathway activation by mitochondrial FAO inhibition remain unclear.

Interestingly, it has been reported that muscle tissue–specific CPT1b knockout strongly induced an increase in the acetyl-CoA levels in mice muscle (27). Acetyl-CoA is at the crossroad of the metabolism of the three major nutrients and participates in multiple biological processes as a precursor for anabolic reactions, a modulator of enzymatic activity, or a key determinant of protein acetylation (28). In recent years, acetyl-CoA gained more and more attention as a major acetyl group donor for the protein acetylation process. Besides, the activity of the mTOR complex can also be modulated by indirect or direct acetylation. For instance, tuberous sclerosis complex 2 acetylation increased its own ubiquitinated degradation and thus reduced its negative regulatory effect on mTORC1 activity (29). Also, the acetylation of histones H3 and H4 mediated by histone deacetylases (HDACs) can be involved in the activation of the mTORC1 pathway, according to studies in cancer cells (30). In addition, increased intracellular acetyl-CoA levels could promote cell growth and proliferation by inducing histone acetylation (31). And Raptor acetylation was also found to mediate the activation of the mTORC1 pathway (32, 33, 34). Taken together, an elevation of acetyl-CoA levels can regulate mTORC1 through acetylation in different models.

In this study, we used the zebrafish model to explore the consequences of the nutrient metabolism remodeling induced by FAO inhibition on mTORC1 regulation. We further elucidated the underlying mechanisms through *in vivo* and *in vitro* approaches by using MD or etomoxir (specific inhibitor of Cpt-1) as inhibitor of FAO and constructing Cpt-1 knockout fish. We detected the nutrient turnover by isotope tracing and measured the acetyl-CoA production plus the acetylation regulation of mTORC1. Our study revealed that mitochondrial FAO inhibition promoted glucose catabolism and increased acetyl-CoA production, which in turn activated mTORC1 through general control nondepressible 5 (Gcn5)–dependent raptor acetylation.

## Results

### Mitochondrial FAO inhibition activates mTORC1 pathway and increases protein deposition

**Figure 1:**
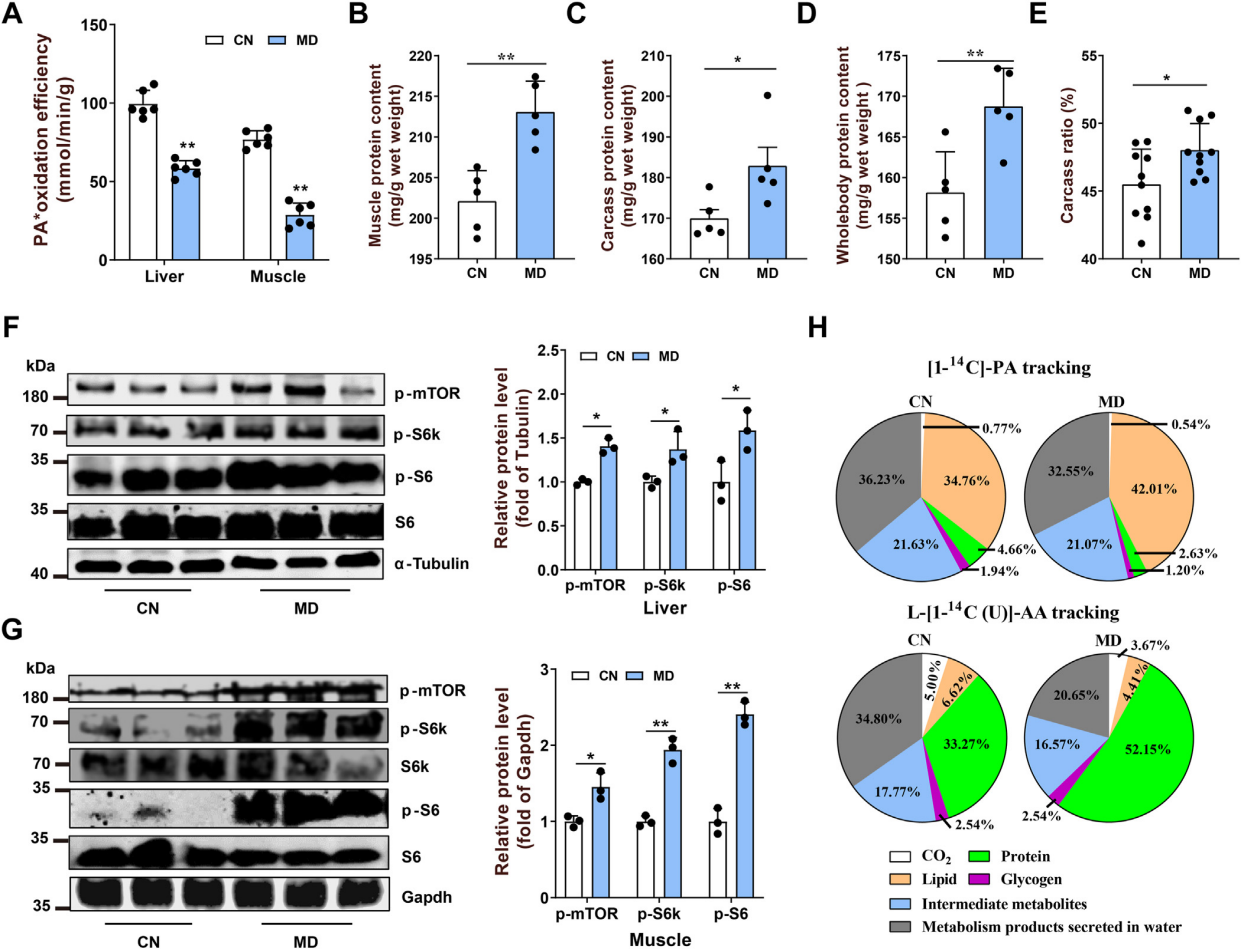
**Mitochondrial FAO inhibition activates the mTORC1 pathway and increases protein synthesis in zebrafish.***A*, isotope tracer analysis (PA∗, [1–14C] palmitic acid) to assess mitochondrial FAO efficiency in liver and muscle tissues. N = 6. *B*–*E*, the effects of mitochondrial FAO inhibition by mildronate (MD) on muscle protein content (*B*, n = 5), carcass protein content (*C*, n = 5), whole body protein content (*D*, n = 5), and carcass ratio (CR) (*E*, n = 9) in zebrafish. CR = 100 × (carcass weight/body weight). The carcass is mainly muscle tissue. *F* and *G*, the mTORC1 activity in liver (*F*) and muscle (*G*) tissues was detected by Western blotting for key proteins of the mTORC1 pathway. p indicates phosphorylated protein. Liver and muscle tissues were lysed, and Western blots for p-mTOR^Ser2448^, p-S6k^Thr389^, S6k, p-S6^Ser235/236^, and S6 are shown. N = 3. *H*, metabolic tracking of [1–14C]-PA and l-[14C (U)]-AA in zebrafish-fed CN and MD diets for 6 weeks (*H*). N = 6. Data represent mean ± SD. ∗*p* < 0.05 and ∗∗*p* < 0.01. AA, amino acid; CN, control; FAO, fatty acid oxidation; mTORC1, mechanistic target of rapamycin complex 1.

**Supplemental Figure S1:**
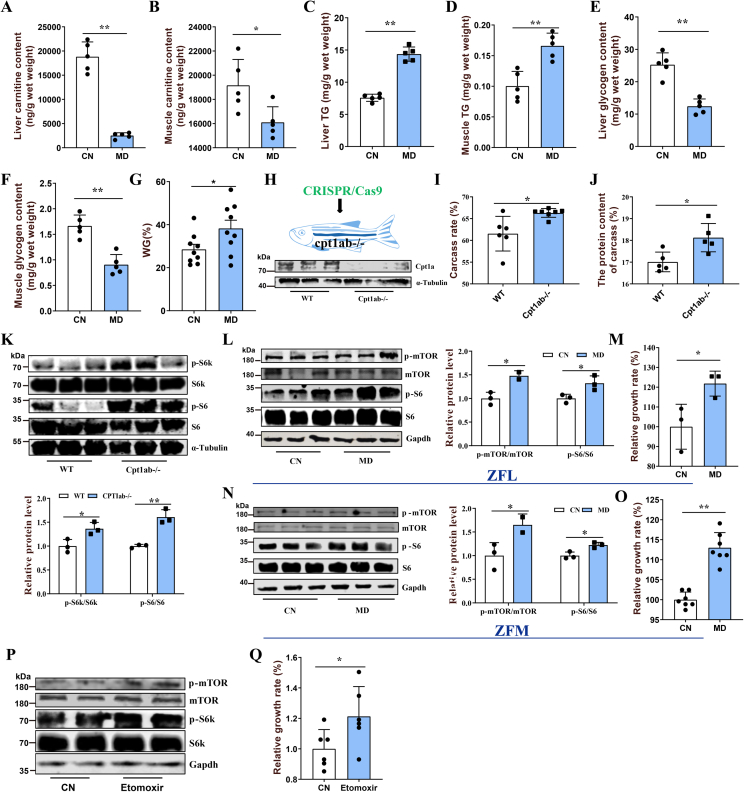


The 6-week MD feeding inhibited the mitochondrial β-oxidation of [1^14^C]-palmitic acid (PA) in the liver and muscle significantly (Fig. 1*A*), accompanying with a remarkably reduced l-carnitine content (Fig. S1, *A* and *B*), increased fat accumulation (Fig. S1, *C* and *D*), and reduced glycogen contents (Fig. S1, *E* and *F*) in both liver and muscle. Simultaneously, the protein contents of muscle, carcass, and whole fish increased in the MD-fed fish (Fig. 1, *B*–*D*), along with a rise in carcass ratio (Fig. 1*E*) and body weight (Fig. S1*G*). Consistently, the mTORC1 pathway–related p-mTOR, p-S6k, and p-S6 protein levels were increased in both liver and muscle tissues in the MD-treated animal (Fig. 1, *F* and *G*). Similar phenotypes, including carcass ratio, carcass protein content, and p-S6k and p-S6 protein levels, were also found in the *cpt1ab*-deficient fish (*cpt1ab*^*−/−*^) (Fig. S1, *H–K*).

To accurately investigate the metabolic turnover of lipid and protein in the fish with FAO inhibition, we intraperitoneally injected zebrafish individually using ^14^C-labeled PA or ^14^C-labeled AA mixture. As expected, more PA-sourced ^14^C (42.01%) was retained in the lipid fraction of the MD-treated zebrafish than the control (CN; 34.76%) (Fig. 1*H*). After the ^14^C-labeled AA injection, the proportion of the ^14^C retained in protein fraction in the MD-treated fish (52.15%) was also much higher than that in the CN (33.27%) (Fig. 1*H*). *In vitro*, using zebrafish fish liver or muscle cells, we also found that the MD- or etomoxir-induced mitochondrial FAO inhibition significantly activated the mTORC1 pathway and promoted cell proliferation (Fig. S1, *L–Q*). These results suggest that mitochondrial FAO inhibition activated the mTORC1 signaling pathway, promoted protein deposition, and resulted in a mass gain.

### Mitochondrial FAO inhibition–stimulated mTORC1 pathway is associated with the increased glucose-derived acetyl-CoA

**Figure 2:**
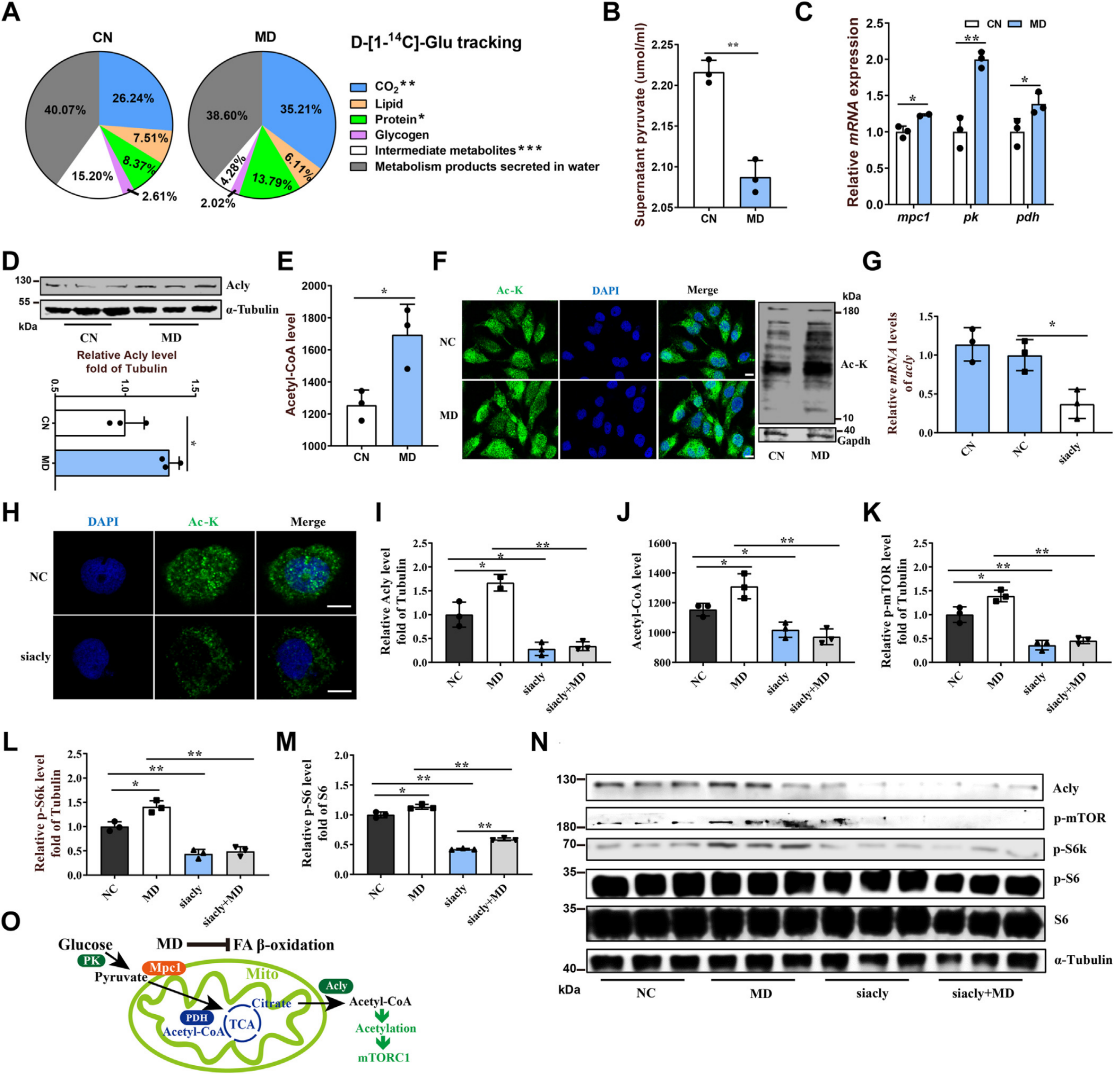
**Mitochondrial FAO inhibition increases cytoplasmic glucose-derived acetyl-CoA levels and protein acetylation–mediated mTORC1 activation.***A*, metabolic tracking of d-[1–14C] glucose in zebrafish-fed CN and MD diets for 6 weeks (*H*). N = 6. *B*, the pyruvate content in ZFL cell culture supernatant. N = 3. The concentration and time of MD-treated cells were 1 mM and 48 h (the same below). *C*, relative mRNA levels of key regulatory genes (*mpc1*, *pk* [pyruvate kinase] and, *pdh* [pyruvate dehydrogenase]) for glucose-derived acetyl-CoA production in ZFL cells. N = 3. *D* and *E*, the protein expression levels of Acly (*D*; N = 3) and intracellular acetyl-CoA levels (*E*; N = 3) in ZFL cells. *F*, immunofluorescence (*left*) and Western blotting (*right*) of the global protein lysine acetylation in ZFL cells. Ac-K indicates protein lysine acetylation. Scale bars represent 10 μm. N = 3. *G*, relative mRNA levels of *acly* (ATP citrate lyase) in CN, NC, and *acly* siRNA-treated cells. N = 3. *H*, immunofluorescence of the global protein lysine acetylation in *acly* siRNA-treated ZFL cells. Scale bars represent 5 μm. N = 3. *I*, the protein expression levels of Acly were quantified after NC and MD cells were treated with *acly* siRNA for 48 h. N = 3. *J*, the intracellular acetyl-CoA levels were quantified after NC and MD cells were treated with *acly* siRNA for 48 h. N = 3. *K*–*M*, the effect of *acly* knockdown on mTORC1 signaling caused by mitochondrial FAO inhibition. NC and MD cells were treated with *acly* siRNA for 48 h. Relative protein quantification of p-mTOR/tubulin (*K*), p-S6k/tubulin (*L*), and p-S6/S6 (*M*) in ZFL cells. N = 3. *N*, the Western blots for Acly, p-mTOR^Ser2448^, p-S6k^Thr389^, p-S6^Ser235/236^, and S6 are shown. N = 3. *O*, mitochondrial FAO inhibition increases glucose-derived acetyl-CoA production, which in turn promotes cytoplasmic protein acetylation-mediated mTORC1 activation. Data represent mean ± SD. ∗*p* < 0.05 and ∗∗*p* < 0.01. Acly, ATP citrate lyase; CN, control; FAO, fatty acid oxidation; MD, mildronate; mTORC1, mechanistic target of rapamycin complex 1; NC, negative control; ZFL, zebrafish liver.

**Supplemental Figure S2:**
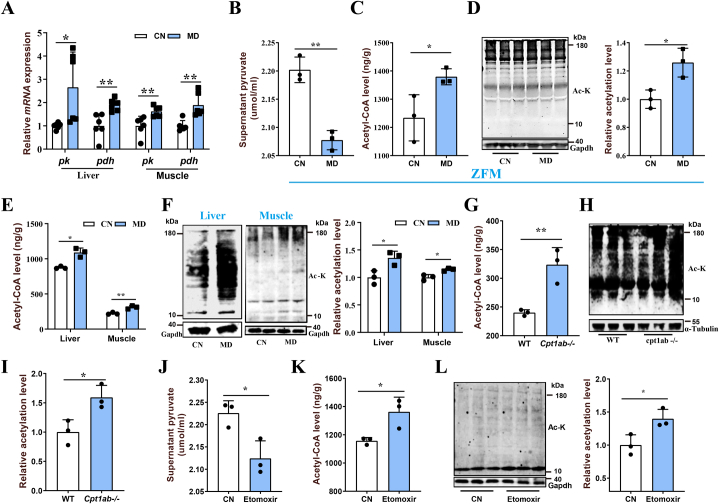


One can speculate that the primary energy source should be glucose catabolism under our conditions of FAO inhibition. After injection of ^14^C-labeled glucose, we found a significantly elevated ^14^C in the released CO_2_ from the MD-treated zebrafish (Fig. 2*A*). Accordingly, glucose catabolic key genes, including pyruvate kinase (*pk*) and pyruvate dehydrogenase (*pdh*), were also significantly upregulated in the liver and muscle of MD-treated zebrafish compared with CN-treated zebrafish (Fig. S2*A*). These results further indicate that MD treatment enhanced glucose catabolism.

Next, we found that the pyruvate content was significantly reduced in the MD-treated zebrafish liver (ZFL) and zebrafish muscle (ZFM) cells (Fig. 2*B* and S2*B*) and in eetomoxir-treated ZFL cells (Fig. S2*J*), whereas the expression of the mitochondrial pyruvate transporter 1 (*mpc1*), *pk*, and *pdh* was upregulated in the MD-treated ZFL (Fig. 2*C*), suggesting an increase in mitochondrial acetyl-CoA production from glucose. Moreover, the protein expression of ATP citrate lyase (Acly), which catalyzes the formation of acetyl-CoA from mitochondria-derived citrate in the cytoplasm, was significantly increased in the MD-treated ZFL cells (Fig. 2*D*). Accordingly, acetyl-CoA concentration and lysine-acetylated protein level (green fluorescent signal and protein expression) were significantly increased in MD-treated ZFL cells (Fig. 2, *E* and *F*), in MD-treated ZFM cells (Fig. S2, *C* and *D*), as well as in the liver and muscle of MD-fed fish (Fig. S2, *E* and *F*), *cpt1ab*^*−/−*^ fish muscle tissue (Fig. S2, *G*–*I*), and etomoxir-treated ZFL cells (Fig. S2, *K* and *L*).

Considering that cellular acetyl-CoA can regulate cell growth and proliferation by promoting protein acetylation (35), we examined if acetyl-CoA could induce the acetylation of mTOR-related proteins. First, we found that *acly* knockdown noticeably inhibited the acetylation level of cytoplasmic proteins (Fig. 2, *G* and *H*, lowered green signal in cytoplasmic area), indicating a reduced cytoplasmic acetyl-CoA level. We further found that the *acly* knockdown significantly lowered the intracellular acetyl-CoA level in ZFL cells with or without MD existence, verifying that mitochondrial FAO inhibition primarily increased the cytoplasmic acetyl-CoA level (Fig. 2, *I* and *J*). Moreover, the phosphorylation of mTORC1 pathway–related proteins (p-mTOR, p-S6k, and p-S6) was also significantly inhibited in the *acly* knockdown ZFL cells regardless of the presence of MD (Fig. 2, *K–N*). These indicate that mitochondrial FAO inhibition might stimulate mTORC1 activation through increasing cytoplasmic glucose-derived acetyl-CoA and protein acetylation (Fig. 2*O*).

### Mitochondrial FAO inhibition stimulates mTORC1 activity *via* Raptor acetylation

**Figure 3:**
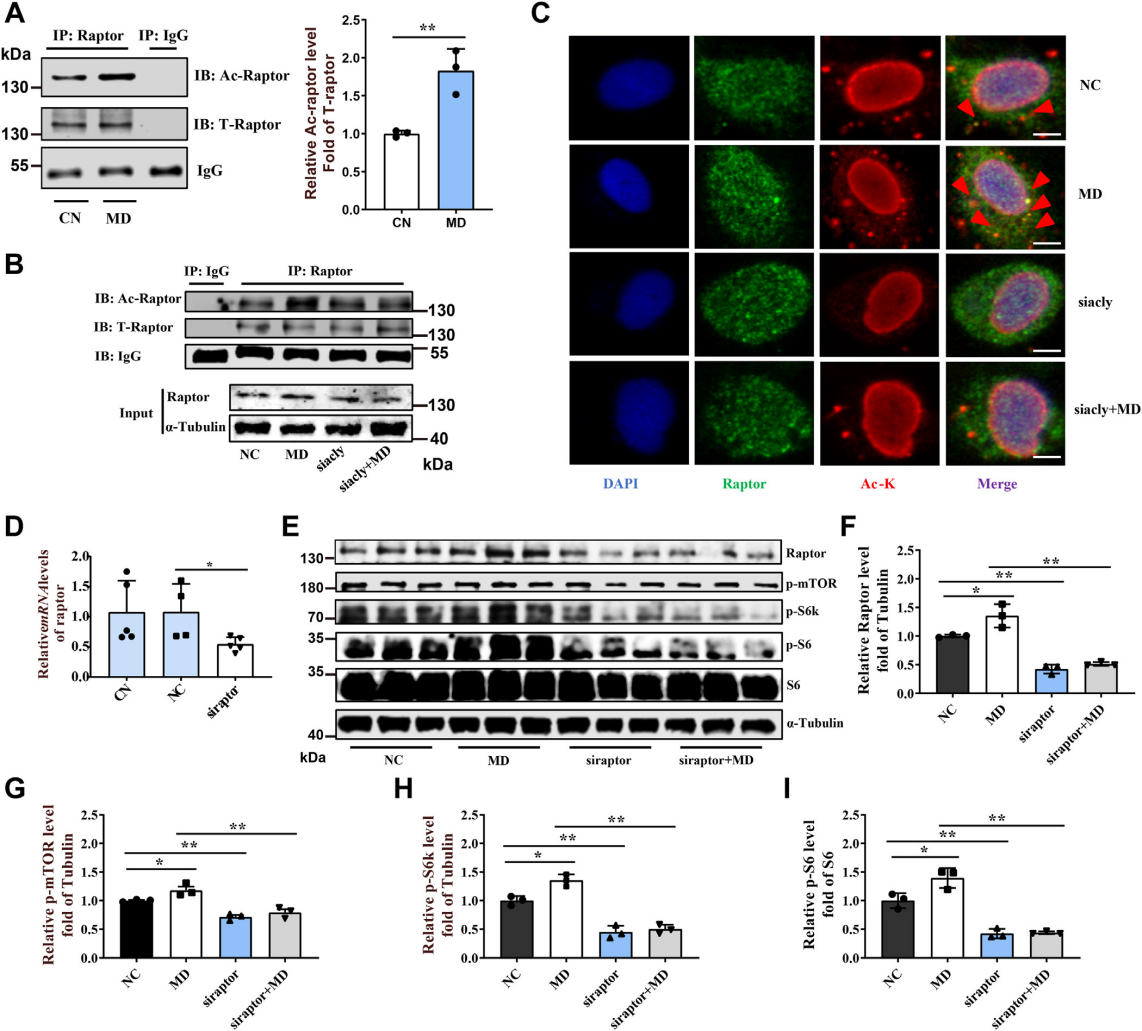
**Mitochondrial FAO inhibition activates mTORC1 activity dependent on Raptor acetylation.***A*, immunoprecipitation of Raptor followed by detection of Raptor acetylation levels with antilysine acetylation antibody in CN- and MD-treated ZFL cells after 48 h. N = 3. *B*, immunoprecipitation of Raptor followed by detection of Raptor acetylation levels with antilysine acetylation antibody. The NC, MD, *acly* siRNA, and MD with *acly* siRNA–treated cells were lysed and Western blots for Raptor acetylation. N = 3. *C*, subcellular localization of Raptor and lysine-acetylated proteins in NC, MD, *acly* siRNA, and MD with *acly* siRNA–treated cells after 48 h. Scale bars represent 5 μm. *D*, relative mRNA levels of *Raptor* in CN, negative control (NC), and *raptor* siRNA–treated ZFL cells. N = 5. *E*–*I*, the effect of *raptor* knockdown on mTORC1 signaling caused by mitochondrial FAO inhibition. NC and MD cells were treated with *raptor* siRNA for 48 h. Relative protein quantification of Raptor/tubulin (*F*), p-mTOR/tubulin (*G*), p-S6k/tubulin (*H*), and p-S6/S6 (*I*) in ZFL cells. Cells were lysed, and Western blots for Raptor, p-mTOR^Ser2448^, p-S6k^Thr389^, p-S6^Ser235/236^, and S6 are shown (*E*). N = 3. Data represent mean ± SD. ∗*p* < 0.05 and ∗∗*p* < 0.01. Ac-Raptor, lysine acetylation of Raptor; CN, control; FAO, fatty acid oxidation; MD, mildronate; mTORC1, mechanistic target of rapamycin complex 1; NC, negative control; T-Raptor, total Raptor; ZFL, zebrafish liver.

**Supplemental Figure S3:**
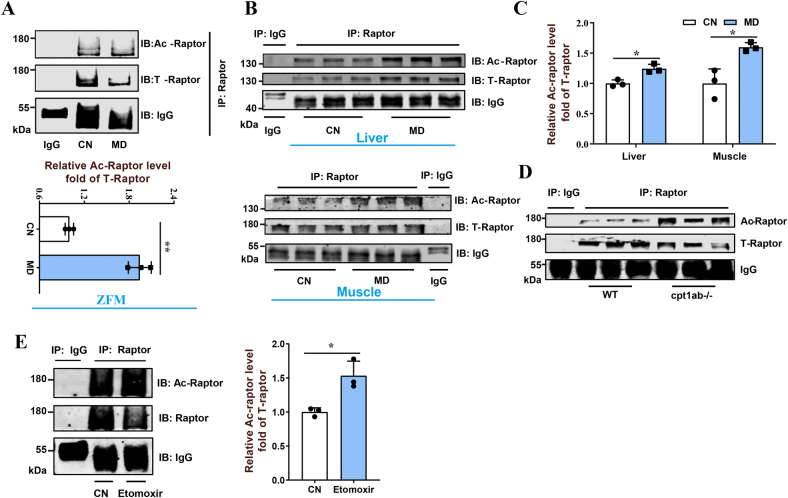


The present study also found that mitochondrial FAO inhibition promoted Raptor acetylation in ZFL cells (Fig. 3*A*), ZFM cells (Fig. S3*A*), liver and muscle tissues of MD-fed fish (Fig. S3, *B* and *C*), and *cpt1ab*^*−/−*^ fish muscle tissue (Fig. S3*D*). Interestingly, *acly* knockdown inhibited the increase of Raptor acetylation induced by MD treatment in ZFL cells, as demonstrated by Western blot analysis (Fig. 3*B*) and subcellular localization of Raptor acetylation, since *acly* knockdown noticeably reduced the yellow colocalization fluorescence signal (representing Raptor acetylation) (Fig. 3*C*). Therefore, to determine whether Raptor mediates the activation of mTORC1 induced by mitochondrial FAO inhibition, we knocked down *raptor* by transfecting *raptor* siRNA into ZFL cells (Fig. 3*D*). The *raptor* knockdown reduced the protein levels of p-mTOR, p-S6k, and p-S6 significantly in ZFL cells and fully abrogated the effect of MD treatment (Fig. 3, *E*–*I*). Together, these results show that mitochondrial FAO inhibition activated mTORC1 *via* Raptor acetylation.

### Mitochondrial FAO inhibition–induced acetylation of Raptor requires the involvement of acetyltransferase Gcn5

**Figure 4:**
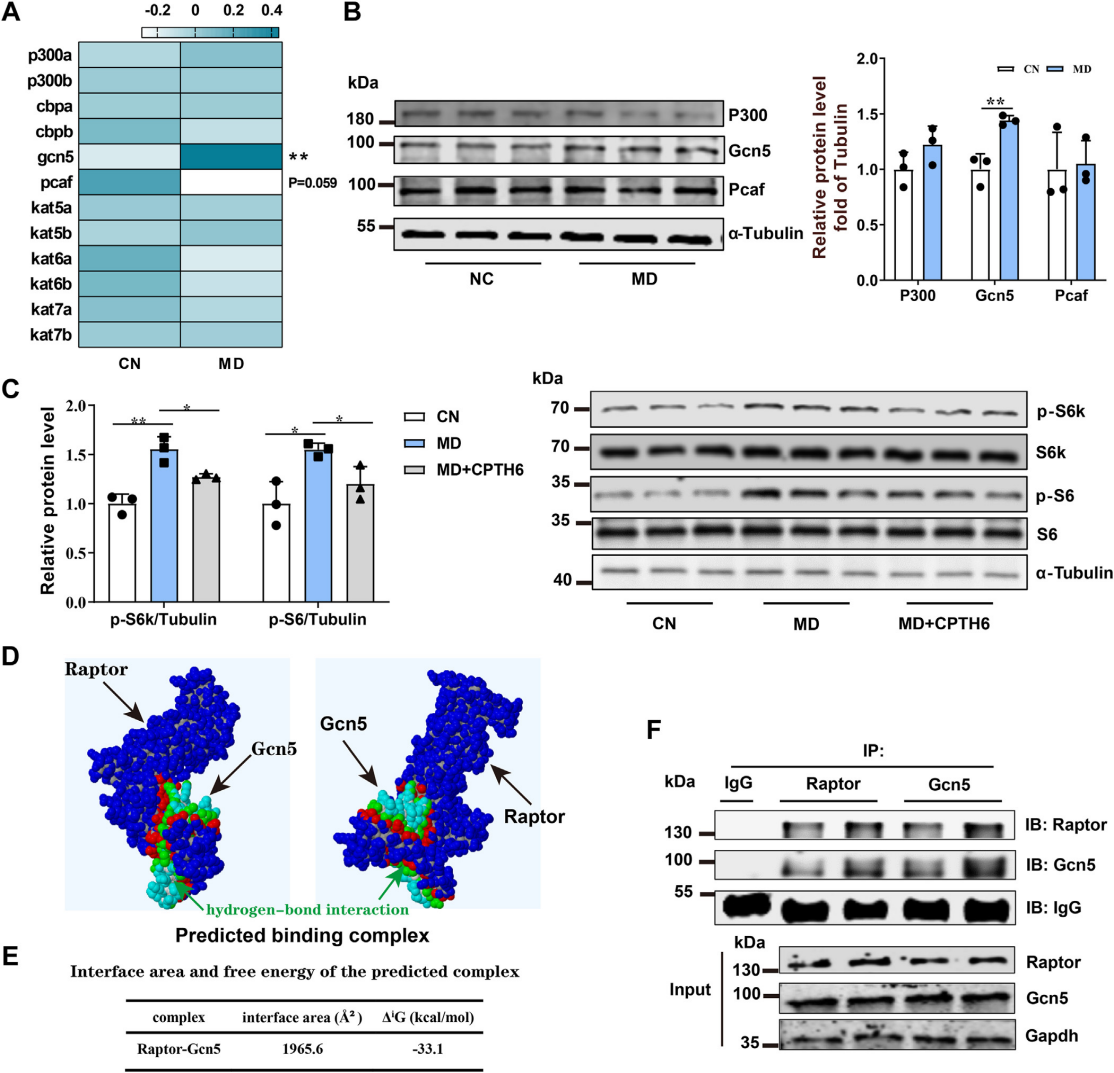
**Mitochondrial FAO inhibition activates the mTORC1 pathway dependent on the acetyltransferase Gcn5.***A*, the effects of mitochondrial FAO inhibition on mRNA expression levels of acetyltransferase family related genes in ZFL cells. As shown in the heat map. N = 5. *B*, the effects of mitochondrial FAO inhibition on the protein expression levels of key acetyltransferases P300, Gcn5, and Pcaf in ZFL cells. N = 3. *C*, the effect of Gcn5 inhibitor (CPTH6, 1 μM) treatment on mTORC1 activity (p-S6k^Thr389^ and p-S6^Ser235/236^) caused by mitochondrial FAO inhibition. The CN- (normal medium), MD- (normal medium containing 1 mM MD), and MD + CPTH6- (normal medium containing 1 mM MD and 1 μM CPTH6) treated cells were lysed and Western blotted after 48 h. N = 3. *D*, the 3D spatial structures of zebrafish Gcn5 and Raptor were obtained from SWISS-MODE and the predicted binding complex model of Raptor–Gcn5 by using the PDBePISA online protein docking tool. Note: different atoms are marked with different colors, *gray* is hydrogen, *green* is carbon, *red* is oxygen, and *blue* is nitrogen. *E*, the interface area and free energy of the predicted complex was analyzed and shown in the table. The larger the interface area implies the easier the proteins bind to each other. Negative free energy indicates that the protein can bind stably. *F*, immunoprecipitation reflects the protein interactions between Raptor and Gcn5 in ZFL cells. Data represent mean ± SD. ∗*p* < 0.05 and ∗∗*p* < 0.01. CN, control; FAO, fatty acid oxidation; Gcn5, general control nondepressible 5; MD, mildronate; mTORC1, mechanistic target of rapamycin complex 1; Pcaf, P300/Creb-binding protein–associated factor; ZFL, zebrafish liver.

To explore the exact mechanism by which acetyl-CoA induced Raptor acetylation in low FAO conditions, we checked which lysine acetyltransferases were involved in catalyzing the acetylation of Raptor in ZFL cells. Although the exact number of lysine acetyltransferases is still controversial based on current knowledge, they can be grouped into three major families: Gcn5/Pcaf (P300/Cbp [Creb-binding protein]-associated factor), P300/Cbp, and MYST (Kat5, Kat6, Kat7, and Kat8) (36). Therefore, we first evaluated the gene expression of members from the three acetyltransferase families mentioned previously in the MD-treated ZFL cells. The results showed that although the gene expression level of *pcaf* was downregulated, its protein expression remained stable (Fig. 4, *A* and *B*). Besides, neither gene expression nor protein expression of P300 was altered significantly, although P300 has been reported to catalyze the acetylation of Raptor (33, 34, 37) (Fig. 4, *A* and *B*). However, the *mRNA* and protein expression of *Gcn5* was markedly upregulated (Fig. 4, *A* and *B*). Accordingly, we found that CPTH6, a specific inhibitor of Gcn5, was able to reduce the protein levels of p-S6k and p-S6 in the MD-treated ZFL cells (Fig. 4*C*), indicating Gcn5 did play a role in the regulation of the mTORC1 pathway in low FAO conditions.

Subsequently, we predicted the 3D complex model of Raptor and Gcn5 and their potential interaction domains using a protein–protein docking approach. The 3D spatial structures of Raptor and Gcn5 revealed that they could interact and form a complex mainly through hydrogen bonds (Fig. 4*D*). In addition, the binding free energy between Raptor and Gcn5 is low, indicating that the Raptor–Gcn5 complex is very stabilized (Fig. 4*E*). Furthermore, the immunoprecipitation analysis confirmed the interaction between Gcn5 and Raptor proteins in ZFL cells (Fig. 4*F*), indicating that Gcn5 could directly catalyze Raptor acetylation.

### Gcn5 is the main acetyltransferase in mediating raptor acetylation regulation

**Figure 5:**
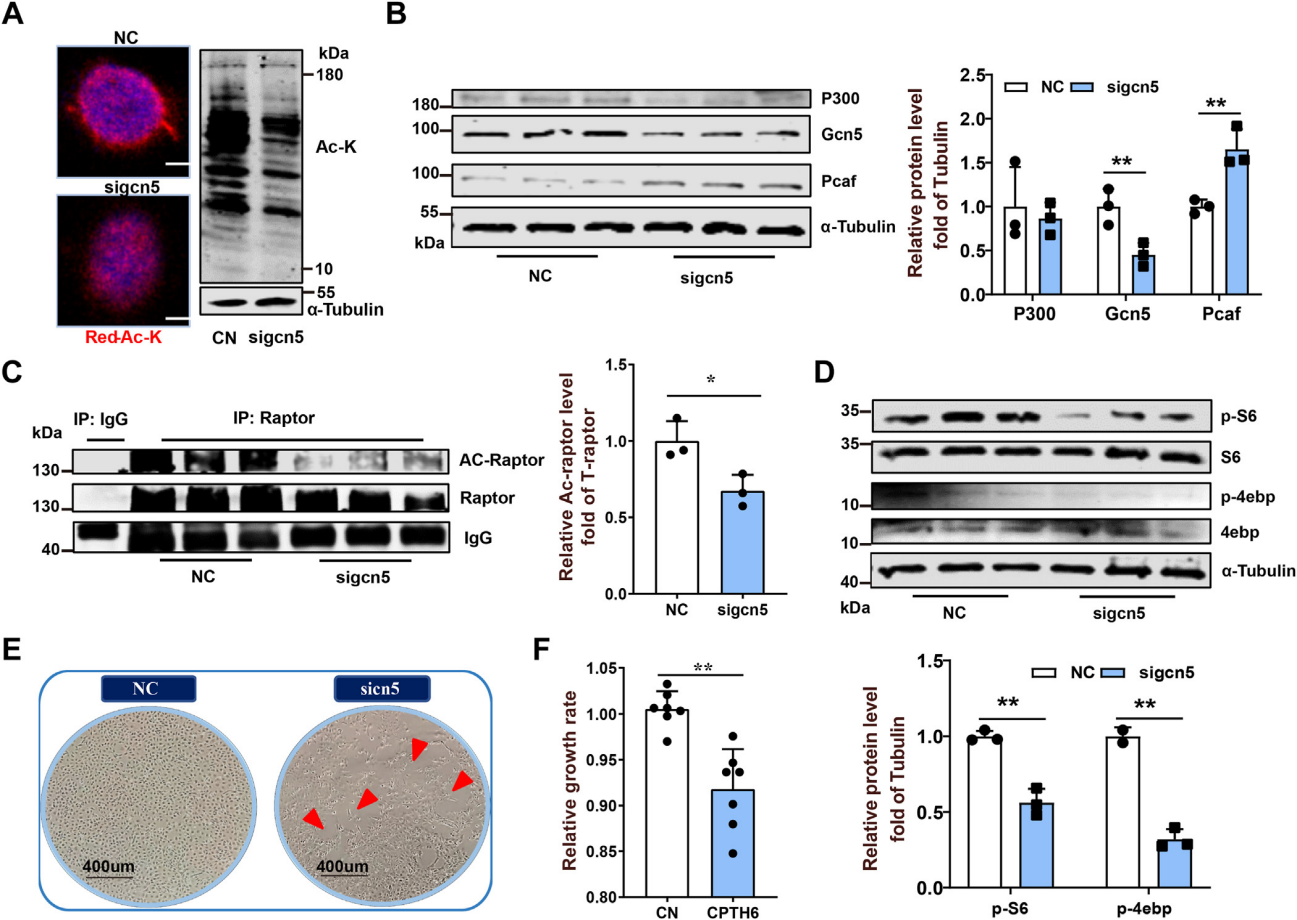
**Acetyltransferase Gcn5 mediates the regulation of Raptor acetylation in ZFL cells.***A*–*C*, the effects of Gcn5 siRNA treatment on global protein lysine acetylation (*A*), protein quantification of acetyltransferase (P300, Gcn5, and Pcaf) (*B*), and Raptor acetylation (*C*). Scale bars represent 5 μm. N = 3. *D*, the effects of *gcn5* siRNA treatment on mTORC1 activity (p-S6^Ser235/236^ and p-4EBP^Thr37/46^) in ZFL cells. N = 3. *E*, the effect of *gcn5* siRNA treatment on cell growth performance in ZFL cells. N = 3. *F*, the effect of CPTH6 treatment on the relative growth rate of ZFL cells. N = 7. Data represent mean ± SD. ∗*p* < 0.05 and ∗∗*p* < 0.01. Gcn5, general control nondepressible 5; mTORC1, mechanistic target of rapamycin complex 1; Pcaf, P300/Creb-binding protein–associated factor; ZFL, zebrafish liver.

**Supplemental Figure S4:**
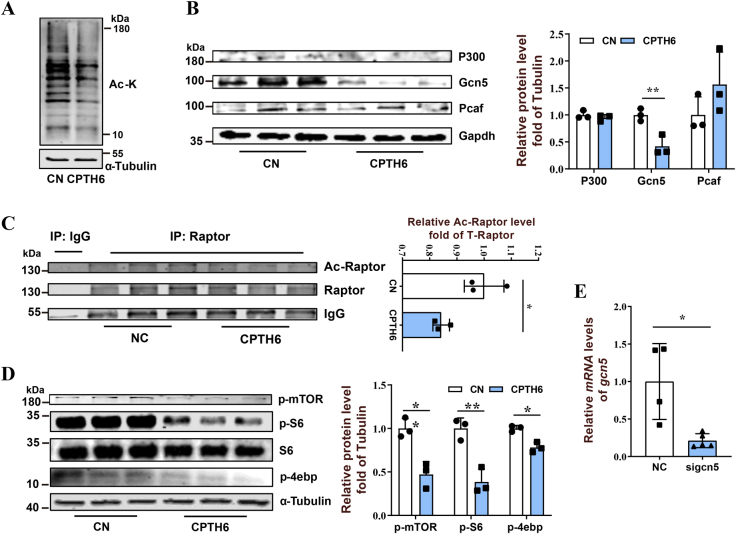


**Supplemental Figure S5:**
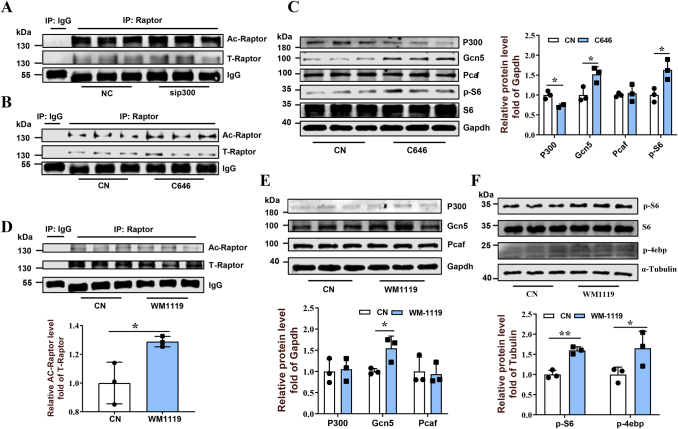


To further confirm that Gcn5 mediates Raptor acetylation, we used *gcn5* siRNA and CPTH6 to inhibit Gcn5 in ZFL cells. It turns out that both treatments decreased the global protein lysine acetylation, Gcn5 protein level (Figs. 5, *A* and *B* and S4, *A* and *B*), and Raptor acetylation in ZFL cells (Figs. 5*C* and S4*C*). Accordingly, the levels of p-S6 and p-4ebp were also repressed in the *gcn5* siRNA or CPTH6-treated ZFL cells, revealing the suppression of the mTORC1 pathway activity (Figs. 5*D* and S4*D*). Moreover, cell growth was inhibited by the functional loss of Gcn5 as the proliferation and growth rates of *gcn5* siRNA- or CPTH6-treated ZFL cells lowered (Fig. 5, *E* and *F*). In addition, both *P300* siRNA and P300-specific inhibitor C646 treatment did not reduce Raptor acetylation in ZFL cells (Fig. S5, *A* and *B*). The C646 treatment reversely increased Gcn5 expression (Fig. S5*C*), Raptor acetylation (Fig. S5*B*), and p-S6 level (Fig. S5*C*) in ZFL cells. Similar results were also observed in the ZFL cells treated with the MYST acetyltransferase–specific inhibitor WM1119 (Fig. S5, *D–F*). Furthermore, the Gcn5-specific inhibitor CPTH6 markedly blocked mTORC1 activation (p-S6 protein level) in C646- and WM1119-treated ZFL cells (Fig. S6, *A* and *B*). Altogether, these results confirm that activation of mTORC1 by Raptor acetylation mainly depends on Gcn5 but not P300 and MYST acetyltransferase.

### Deacetylase HDACs mediated raptor acetylation and mTORC1 pathway regulation

**Figure 6:**
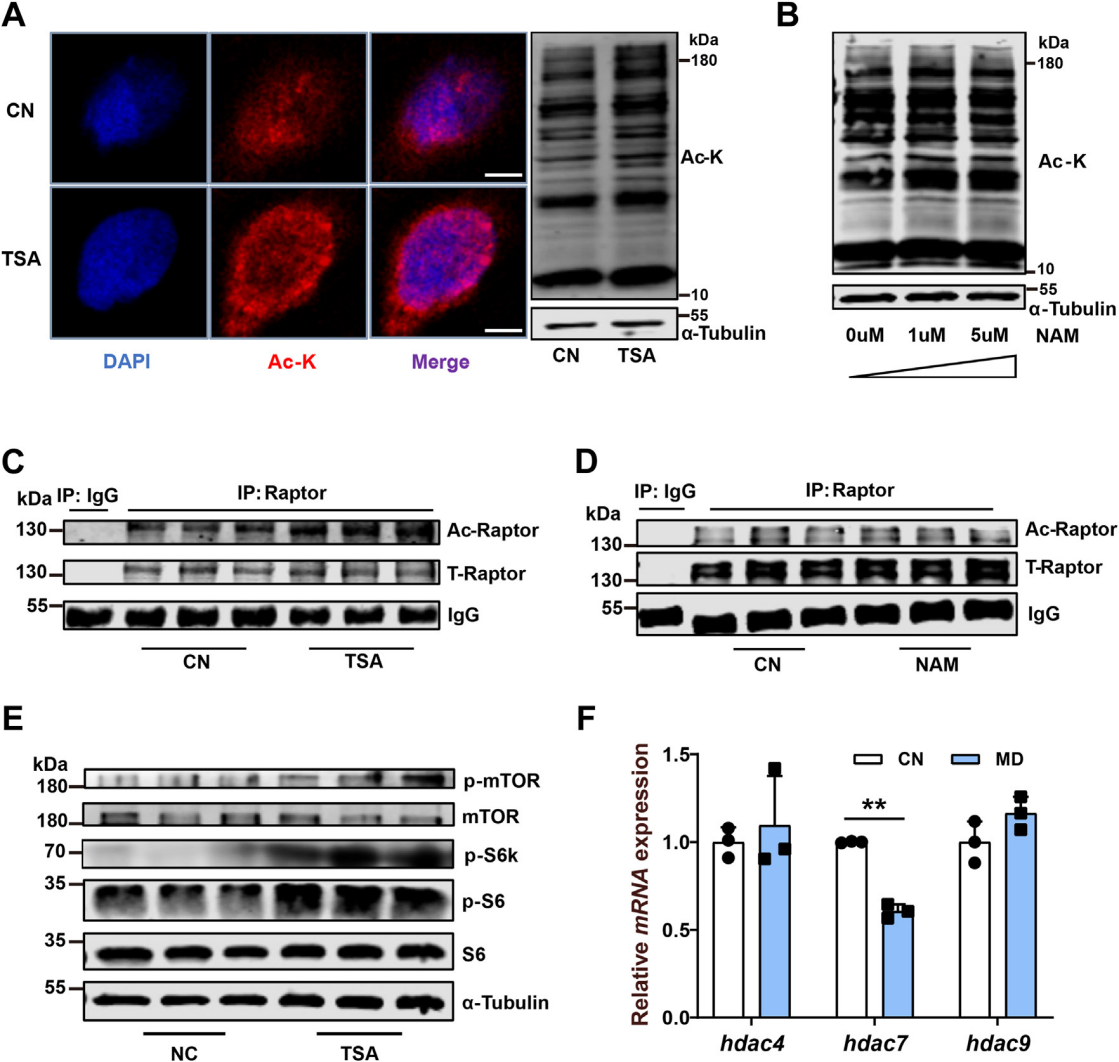
**Deacetylase HDAC mediates Raptor acetylation and mTORC1 pathway regulation.***A*, immunofluorescence and Western blotting of the global protein lysine acetylation in CN and HDAC inhibitor (trichostatin A [TSA]: 10 μM) treated ZFL cells. Scale bars represent 5 μm. N = 3. *B*, the effects of deacetylase SIRT inhibition (nicotinamide [NAM]: 0, 1, and 5 μM) on global protein lysine acetylation in ZFL cells. N = 3. *C*, Raptor acetylation in CN- and TSA-treated ZFL cells. N = 3. *D*, Raptor acetylation in CN- and NAM- (5 μM) treated ZFL cells. N = 3. *E*, mTORC1 activity (p-mTOR^Ser2448^, p-S6k^Thr389^, and p-S6^Ser235/236^) in CN- and TSA-treated ZFL cells. N = 3. *F*, the effects of mitochondrial FAO inhibition (MD treatment) on mRNA expression levels of class IIa HDAC-related genes in ZFL cells. N = 3. Data represent mean ± SD. ∗*p* < 0.05 and ∗∗*p* < 0.01. CN, control; HDAC, histone deacetylase; MD, mildronate; mTORC1, mechanistic target of rapamycin complex 1; SIRT, sirtuin deacetylase; ZFL, zebrafish liver.

Protein lysine acetylation relies on the coordinated action of lysine acetyltransferases and lysine deacetylases. The deacetylases can be divided into two major categories: Zn^2+^-dependent HDACs and NAD^+^-dependent sirtuin deacetylases (SIRTs) (38). To fully understand the regulation of Raptor acetylation, we selected trichostatin A (TSA) and nicotinamide, specific inhibitors of HDACs and SIRTs, respectively, to treat ZFL cells. Both TSA and nicotinamide increased global protein lysine acetylation remarkably (Fig. 6, *A* and *B*), but only TSA increased Raptor acetylation (Fig. 6, *C* and *D*), showing that Raptor deacetylation was mediated by HDACs rather than SIRTs. In addition, the increased Raptor acetylation was also accompanied by mTORC1 pathway activation in the TSA-treated ZFL cells (Fig. 6*E*).

We then checked the expression of class IIa HDAC-related genes (*hdac*4, 7, and 9) in the MD-treated ZFL cells, since they are the main HDACs involved in intracellular signaling response regulation compared with other HDACs (38). The result showed that the gene expression of *hdac7* was dramatically suppressed when mitochondrial FAO was inhibited (Fig. 6*F*). In summary, HDACs and Gcn5 are jointly involved in Raptor acetylation regulation, and Hdac7 could serve as an important HDAC in regulating Raptor deacetylation under mitochondrial FAO inhibition.

## Discussion

The mTORC1 pathway is considered an essential regulator of either organ hypertrophy or atrophy through regulating protein synthesis (39, 40). Besides, mitochondrial FAO inhibition could activate mTORC1 to increase the synthesis of AAs and proteins, thus often associated with cell proliferation and organ hypertrophy (15, 16, 18, 19, 25). However, the underlying mechanisms have not been illustrated. In a previous study conducted in the zebrafish model, we observed that MD treatment stimulates mTORC1 activation and protein synthesis, whereas rapamycin blocks these effects as an inhibitor of mTORC1 (25). These findings suggest that the mTORC1 pathway is involved in the protein synthesis induced by mitochondrial FAO inhibition. Acetyl-CoA is not only the product of multiple catabolic reactions but also one of the central substrates for anabolism. The intracellular acetyl-CoA abundance and bioavailability reflect the overall cellular energy status. In most cells, acetyl-CoA is mainly derived from various metabolic circuitries in the mitochondrial matrix, namely pyruvate oxidative decarboxylation, FA β-oxidation, and AA catabolism (branched-chain AAs and glutamine) (41, 42). In our zebrafish model that inhibited mitochondrial FAO by MD, acetyl-CoA levels were increased and mainly derived from glucose catabolism, as confirmed by ^14^C-labeled nutrient tracking experiments. Likewise, mitochondrial FAO inhibition also increased acetyl-CoA levels in mouse muscle tissue significantly (27). In line with this, the major metabolic characteristic of mammalian cardiac hypertrophy (associated with physiological/mitochondrial FAO inhibition) is also a diminished mitochondrial FAO and a shift toward enhanced glucose catabolism for ATP production and anabolic substrate supply (15, 16).

Acetyl-CoA is the acetyl donor for protein acetylation that modifies the activity of key regulatory proteins, which manipulate cellular physiological processes. Thus, acetyl-CoA is also considered a second messenger coordinating cellular anabolism and catabolism (43). In general, protein acetylation is controlled by the combined activity of acetyltransferases and deacetylases, which depends on the availability of acetyl-CoA in the corresponding cellular compartment (44). Studies have reported that acetyltransferase P300 can acetylate Raptor with cytoplasmic acetyl-CoA to activate mTORC1 and thereby inhibit autophagy (33, 34, 37). In the present study, we observed that intracellular acetyl-CoA content increases, and the enhancement of glucose breakdown induced by mitochondrial FAO inhibition could activate mTORC1 efficiently *via* Raptor acetylation. However, P300 was not involved in Raptor acetylation in our mitochondrial FAO-inhibited models. Similarly, in mouse embryonic fibroblast cells and human embryonic kidney 293T cells, P300 also did not acetylate Raptor or regulate mTORC1 activity, suggesting that the acetylating effect of P300 may be species and/or cell type dependent (32, 33).

The present findings indicate that Gcn5 plays a more important role than P300 and MYST in regulating Raptor acetylation and mTORC1 in zebrafish. Gcn5 is conserved in eukaryotic species (45) and regulates various biological events, including gene expression, cell proliferation, metabolism, and inflammation (46). Most previous studies on Gcn5 mainly focused on histone acetylation and concomitant transcriptional regulation (46). However, increasing reports suggest that Gcn5 regulates diverse other cellular functions by acetylating nonhistone substrates (47). In the present study, we screened different acetyltransferases and found that Gcn5 mediates Raptor acetylation and regulates mTORC1 activity in ZFL cells. Furthermore, another study indicated that intracellular acetyl-CoA, as a metabolic signal, stimulates cell growth and proliferation by manipulating Gcn5 (31). The important role of Gcn5 in controlling the maturation and size of chondrocytes has also been described in zebrafish (48). Structurally, the surface of Gcn5 has two roughly orthogonal troughs where acetyl-CoA bounds to one cleft and the lysine-containing peptide bounds to the other cleft (49). Indeed, the present findings show a direct interaction between Gcn5 and Raptor, suggesting that Gcn5 can directly catalyze Raptor acetylation. Moreover, Gcn5 can acetylate its substrates in tune with acetyl-CoA fluctuations *in vivo* (31), consistent with the fact that acetyl-CoA is considered a potent regulator of acetyltransferase activity (50).

The Zn^2+^-dependent HDACs share a highly conserved deacetylase domain and are commonly referred to as classical HDACs. Based on their sequence similarities and phylogenetic conservation, the classical HDACs have been classified into class I, class IIa, class IIb, and class IV (51). Among them, class IIa HDACs (HDAC4, 5, 7, and 9), as signal-responsive HDACs, mainly localized in the nucleus and exported to the cytoplasm upon signal activation (38). Our results provide direct evidence that HDACs mediate deacetylation modification of Raptor during mitochondrial FAO inhibition. Meanwhile, a previous study in mice showed that HDAC5 could deacetylate Raptor to regulate mTORC1 (52), but a similar mechanism was not found in HeLa cells (32). Our finding suggested that class IIa HDACs may be crucial in regulating Raptor acetylation, but the mechanism is species and cell type specific. Therefore, the effect of the dynamic balance of acetylation and deacetylation on nutrient metabolism should receive greater attention.

Although mTORC1 is usually associated with AAs and protein metabolism, the present study brings up the fact that lipid metabolism can impact mTORC1 regulation by influencing glucose metabolism. Aside from indicating the complexity and diversity of the mTORC1 regulatory mechanisms, such a result also highlights the importance of glucose catabolism in systemic nutrient metabolism and transformation when lipid catabolism is reduced. In the current study, we found that the glucose-sourced [^14^C] was also involved in the synthesis of protein (Fig. 2*A*), which reveals that the elevated glucose catabolism upon mitochondrial FAO inhibition not only activates mTORC1 by providing more acetyl-CoA for Raptor acetylation but also directly participates in protein synthesis. Actually, glucose catabolism also provides branch point metabolites for several biosynthetic pathways that are essential for the synthesis of nucleotides, nucleotide sugars, AAs, and glycerophospholipids, all of which are necessary for cell proliferation (53). For example, the development of cardiac hypertrophy requires increased glucose consumption to support aspartate synthesis, which drives the increase of biomass (16). Therefore, the shift from FA to glucose catabolism eventually resulted in biomass increase and protein deposition in two main ways: Raptor acetylation–mediated mTORC1 activation and metabolic intermediate supply for AAs and protein synthesis. Also, considering that branched-chain AAs, especially leucine, can activate mTORC1 through pathways including Raptor acetylation or leucine sensors (32, 54, 55, 56). Thus, mitochondrial FAO inhibition might also participate in mTORC1 regulation through AA metabolism, which likewise deserves attention in future studies.

**Figure 7:**
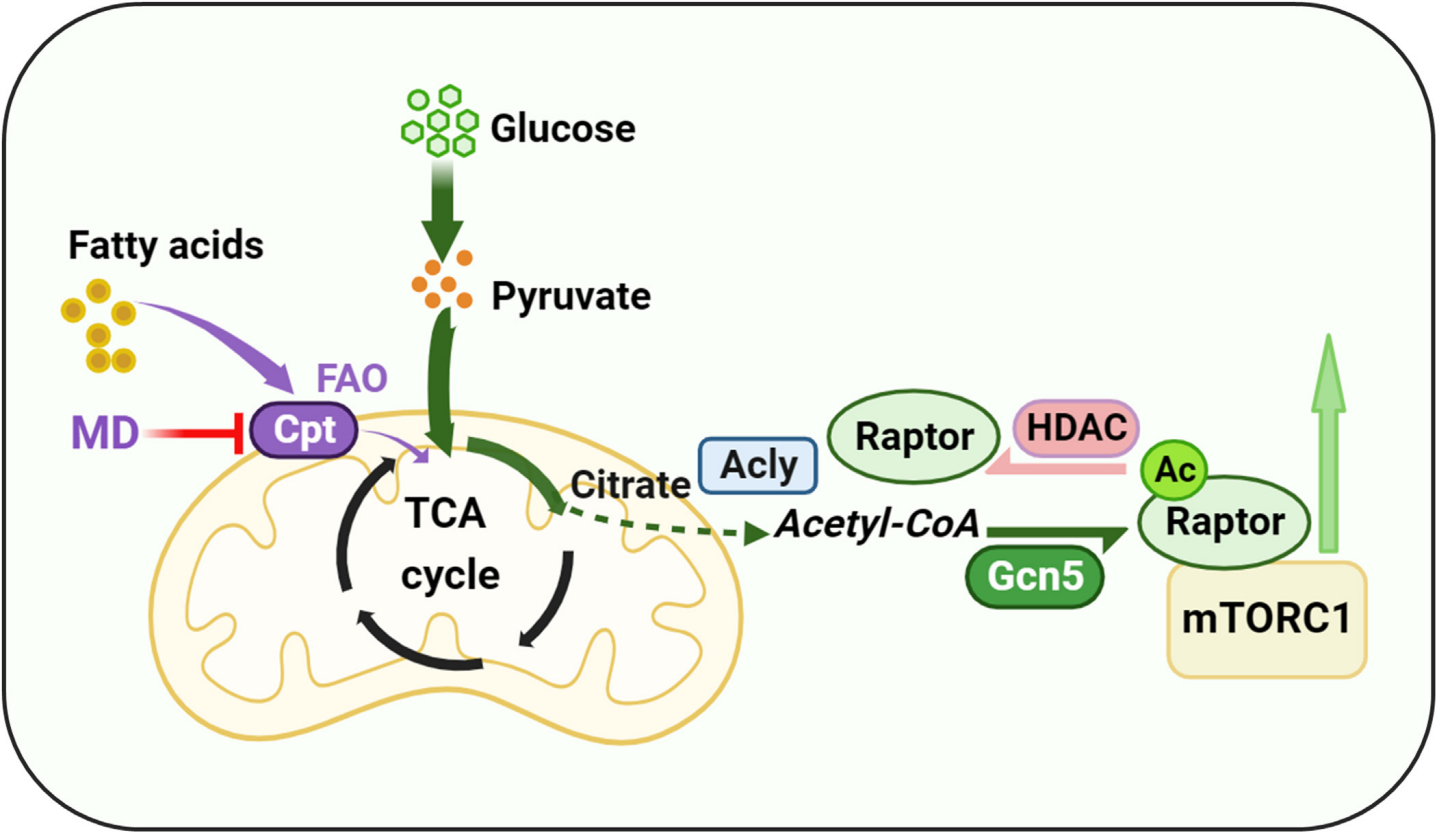
**Schematic model: mitochondrial fatty acid β-oxidation inhibition activates the mTORC1 pathway by promoting glucose catabolism and subsequent Gcn5-dependent Raptor acetylation.** Ac, acetyl; Gcn5, general control nondepressible 5; mTORC1, mechanistic target of rapamycin complex 1; TCA, tricarboxylic acid.

In conclusion, our study indicated that the mitochondrial FAO inhibition induces a compensatory stimulation of glucose catabolism accompanied by an elevation of acetyl-CoA cellular concentration, which enhanced acetylation of Raptor and activated the mTOR pathway to increase protein synthesis and cause cell proliferation/organ hypertrophy. Raptor acetylation induced by increased intracellular acetyl-CoA levels depends on the upregulation of acetyltransferase Gcn5 and the downregulation of deacetylase class IIa HDACs (especially HDAC 7) but was not related to P300 or MYST and SIRTs. These findings provide evidence for an acetyl-CoA-mediated mTORC1 activating mechanism *via* Raptor acetylation in the nutrient metabolism remodeling and enhance understanding of the regulatory role of mitochondrial FAO in systemic physiological and pathological processes. The relevant mechanisms of this study have been summarized in Figure 7.

## Experimental procedures

### Animals

Animal studies were approved by the Committee on the Ethics of Animal Experiments of East China Normal University (F20190101). We conducted all experiments according to the principles of the Laboratory Animal Management guidelines of China. Male zebrafish (360 fish) were kept in a water temperature–controlled (28 °C ± 1.5 deg) environment with a 12:12 h light:dark cycle. Zebrafish weighing approximately 0.47 g were randomly assigned to receive either the CN diet (total 180 fish, n = 3) or the CN supplemented 1 g/kg MD (total 180 fish, n = 3) for 4 weeks. Detailed diet formulation is shown in Table S1. We used CRISPR–Cas9 gene-editing technology to obtain a *cpt1ab* knockout (*cpt1ab*^*−/−*^) zebrafish strain described by our previous work (57). Based on the zebrafish *CPT1ab* genome sequence information, the knockout target was designed as GGGGCCTCGTGGCACAGATGC. The knockout target primers are shown in the Table S2. Under the aforementioned breeding environment, 180 WT zebrafish (n = 3) or *cpt1ab*^*−/−*^ zebrafish (n = 3) (approximately 0.29 g) were fed a CN diet for 4 weeks. All zebrafish should be fasted for 24 h before sampling. After counting the weight gain of zebrafish, the carcass, liver, and muscle were obtained for body index calculation and biochemical analysis.

### Cell culture and transfection

ZFL and ZFM cells were incubated in an L-15 medium with 10% fetal bovine serum and 1% penicillin–streptomycin at 25 °C. ZFL cells were purchased from the China Zebrafish Center. ZFM cells were derived from the ZFM tissue block culture method as previously described (58). Cell proliferative activity was assessed by the Cell Counting Kit-8 assay. siRNA duplexes were transfected using siRNA mate according to the manufacturer's instructions (GenePharma). The specific siRNA primer sequences and Cell Counting Kit-8 kit information are shown in Table S2.

### FAO measurement

Fresh liver and muscle tissue samples from nine zebrafish were weighed and homogenized (1:20, w/v) in ice-cold sucrose medium (0.25 M sucrose, 2 mM EGTA, and 10 mM Tris–Cl, pH 7.4) for FAO measurement. Briefly, the [1-^14^C]-labeled PA was used as a substrate to determine the FAO activity of mitochondria and peroxisomes, as we reported previously (25).

### Western blots and immunoprecipitation

Tissue samples were lysed at 4 °C with lysis buffer (20 mM Tris–HCl [pH 7.4], 5 mM EDTA, 150 mM NaCl, 0.5% Triton X-100, 10 mM sodium butyrate, 1 mM TSA, and protease/phosphatase inhibitor cocktail), and the tissue homogenates were centrifuged to obtain lysates. Cells were lysed using NP-40 buffer (addition 1 mM TSA and protease/phosphatase inhibitor cocktail) and then centrifuged to obtain lysates. The obtained tissue or cell lysates were used for Western blots and immunoprecipitation analysis. The determination of Raptor acetylation in tissues and cells was followed as previously described (32). Briefly, protein samples were separated by SDS-PAGE (New Cell & Molecular Biotech), transferred to nitrocellulose membranes, subjected to Western blot analysis, and finally visualized using Odyssey CLX Imager (LI-COR, Inc) for direct detection of infrared fluorescence. Quantitative analysis of the Western blots was performed using Image Studio Lite software (LI-COR, Inc). Specific information on the antibodies used in this study is provided in Table S2.

### Metabolic tracking of FAs, glucose, and AAs

Before starting the metabolic tracking experiment, zebrafish need to fast for 12 h. Next, fasted zebrafish were injected intraperitoneally with [1-^14^C]-PA, D-[1-^14^C]-Glu, or a mixture of ^14^C evenly labeled l-AA (0.05 μCi/g body weight; PerkinElmer), then ^14^C-CO_2_ was collected in KOH solution, and fat, glycogen, and protein were extracted from whole fish for the ^14^C detection, as in our previous study (25). Fat, glycogen, and protein extraction were conducted as described previously (59, 60). Afterward, the radioactivity of KOH solution and ^14^C-labeled nutrients dissolved in 0.5 ml of lysis solution (30% H_2_O_2_:HClO_4_, 1:2, v/v) was detected by Tri-Carb 4910TR Liquid Scintillation Analyzer (PerkinElmer). Finally, we assessed the metabolic pathways of nutrients in the body by the ratio of ^14^C in the different fractions.

### Immunofluorescence

For immunostaining, ZFL cells were fixed with 4% paraformaldehyde for 10 min followed by permeabilization (0.5% Triton X-100) and blocking (1% bovine serum albumin). The cells are then incubated with the appropriate primary and secondary antibodies (Table S2). The secondary antibodies goat anti-rabbit Alexa 488 and goat antimouse Alexa 594 were used at 1:10,000 dilution. Imaging was performed with Andor DF505 Confocal Microscope (Andor Technology PLC). Fluorescence intensity and colocalization were measured using ImageJ software (NIH).

### Quantitative real-time PCR

Total RNA was extracted with Trizol (Takara) according to the manufacturer’s protocol. Then total RNA (1 μg) was reverse transcribed to complementary DNA with the cDNA Synthesis Kit (Vazyme). AceQ Universal SYBR qPCR Master Mix (Vazyme) was used to perform quantitative real-time PCR. β-actin and elongation factor 1α were used as housekeeping genes. The primer sequences used in this study are shown in Table S3.

### Metabolites and protein content measurements

The triglyceride, glycogen, and pyruvate contents were determined according to the instructions provided by specific commercial assay kits (Jiancheng Biotech Co) (Table S2). The acetyl-CoA concentrations were determined by using HPLC as described previously (61). Briefly, acetyl-CoA standards need to be run first to determine the standard peak times and to plot the standard curve. The HPLC run was finished in 15 min without any additional time for column re-equilibration between the HPLC runs. For detailed steps on the method of acetyl-CoA concentration measurement, see the previously described (24). The protein content of carcass was determined by Kjeltec 8200 (FOSS) (62).

### Quantification and statistical analysis

Results are presented as means ± SD. Data analysis was conducted by Student’s two-tailed *t* test using GraphPad Prism 7 (GraphPad Software, Inc). Results with *p* < 0.05 represents a statistically significant difference. Statistical significance is presented below: ∗*p* < 0.05 and ∗∗*p* < 0.01.

## Data availability

All data are included in the article.

## Supporting information

This article contains supporting information.

## Conflict of interest

The authors declare that they have no conflicts of interest with the contents of this article.

## Figures and Tables

**Supplemental Figure S6:**