# Supplementary material for: Inhibition of mitochondrial fatty acid β-oxidation activates mTORC1 pathway and protein synthesis via Gcn5-dependent acetylation of Raptor in zebrafish
Source: J Biol Chem. 2023 Sep 3;299(10):105220. doi: 10.1016/j.jbc.2023.105220 (PMC10540046; doi:10.1016/j.jbc.2023.105220)
Supplement: Supplemental Table S1 [file mmc1.docx]

**Table S1**. Formulation and proximate composition (dry matter basis) of the experimental diets.

| Ingredients (%) | CN | MD |
| --- | --- | --- |
| Casein  Gelatin  Soybean oil  Corn starch  Mildronate  Vitamin premix^1^  Mineral premix^2^  Ca(H_2_PO_4_)_2_  CMC  Cellulose  DMPT  Choline chloride  BHT  Total  Proximate composition (% dry matter basis)  Dry matter (%)  Crude protein (%)  Crude lipid (%)  Ash (%) | 320  80  60  300  0  10  30  15  30  148.75  1  5  0.25  1000  90.37  38.26  5.26  3.15 | 320  80  60  300  1  10  30  15  30  147.75  1  5  0.25  1000  91.28  38.13  5.32  3.24 |

1 Vitamin premix (mg or IU/kg): 500,000 I.U. (international units) Vitamin A; 50,000 I.U. Vitamin D_3_; 2500 mg Vitamin E; 1000 mg Vitamin K_3_; 5000 mg Vitamin B_1_; 5000 mg Vitamin B_2_; 5000 mg Vitamin B_6_; 5000 μg Vitamin B_12_; 25,000 mg Inositol; 10,000 mg Pantothenic acid; 100,000 mg Cholin; 25,000 mg Niacin; 1000 mg Folic acid; 250 mg Biotin; 10,000 mg Vitamin C.

2 Mineral premix (g/kg): 314.0 g CaCO_3_; 469.3 KH_2_PO_4_; 147.4 g MgSO_4_·7H_2_O; 49.8 g NaCl; 10.9 g Fe (II) gluconate; 3.12 g MnSO_4_·H_2_O; 4.67 g ZnSO_4_·7H_2_O; 0.62 g CuSO_4_·5H_2_O; 0.16 g KJ; 0.08 g CoCl_2_·6H_2_O; 0.06 g NH_4_ molybdate; 0.02 g NaSeO_3_.
