# Supplementary material for: Inhibition of mitochondrial fatty acid β-oxidation activates mTORC1 pathway and protein synthesis via Gcn5-dependent acetylation of Raptor in zebrafish
Source: J Biol Chem. 2023 Sep 3;299(10):105220. doi: 10.1016/j.jbc.2023.105220 (PMC10540046; doi:10.1016/j.jbc.2023.105220)
Supplement: Supplemental Table S2 [file mmc2.docx]

**Table S2**. Key Resources Table

| **REAGENT or RESOURCE** | **Source** | **Identifier** |
| --- | --- | --- |
| **Antibodies** |  |  |
| Mouse monoclonal anti- Tubulin  Mouse monoclonal anti- GAPDH  Rabbit monoclonal anti- ACSS2  Rabbit polyclonal anti- mTOR  Rabbit polyclonal anti- P-mTOR (Ser2448)  Rabbit polyclonal anti- P-S6  Rabbit polyclonal anti- P-S6k (Thr398)  Rabbit monoclonal anti- S6  Rabbit monoclonal anti- P-S6 (Ser235/236)  Rabbit monoclonal anti- P- 4EBP (Thr37/46)  Rabbit polyclonal anti- 4EBP  Rabbit polyclonal anti- CPT1a  Mouse monoclonal anti- Pan Acetylation  Rabbit polyclonal anti- Acetylated-Lysine  Rabbit monoclonal anti- PCAF  Rabbit monoclonal anti- GCN5  Rabbit monoclonal anti- P300  Rabbit monoclonal anti- IgG  Rabbit monoclonal anti- ACLY  Rabbit monoclonal anti- Raptor  Goat anti-Rabbit IgG (H+L) Cross-Adsorbed Secondary Antibody, Alexa Fluor™ 488  Goat anti-Mouse IgG (H+L) Cross-Adsorbed Secondary Antibody, Alexa Fluor™ 594 | HUABIO  HUABIO  Abways  CST  CST  CST  CST  CST  CST  CST  CST  Proteintech  Proteintech  CST  Abways  CST  CST  Abways  Abcam  CST  Invitrogen  Invitrogen | M1501-180  EM1101150  CY599512  #2972  #2971  #9202  #9209  #2217  #4856  #2855  #9452  15184-1-AP  66289-1-Ig  #9441  CY8354  #3305  #86377  CY5125  ab40793  #48648  A-11008  A-11005 |
| **Reagents** |  |  |
| Mildronate  Etomoxir  [1-^14^C]-PA  D-[1-^14^C]-Glu  [^14^C(U)]-L-AA Mixture  C646  CPTH6  WM1119  Trichostatin A  Nicotinamide  DAPI  Protease inhibitor cocktail  Protease and phosphatase inhibitor cocktail  Deacetylase Inhibitor Cocktail  Sodium butyrate | Micxy Chemical  GLPBIO  PerkinElmer  PerkinElmer  PerkinElmer  GLPBIO  GLPBIO  GLPBIO  MCE  MCE  DAPI  Beyotime  Beyotime  Beyotime  Sigma-Aldrich | 76144-81-5  GC16736  NEC534050UC  NEC042V250UC  NEC850E001MC  GC12733  GC43321  GC18154  HY-15144  HY-B0150  28718-90-3  P1005  P1050  P1112  156-54-7 |
| **Critical Commercial Assays** |  |  |
| Triglyceride kit  Pyruvate kit  Glycogen kit  Cell Counting Kit-8 | JianCheng  JianCheng  JianCheng  GLPBIO | A110  A081  A043  GK10001 |
| **Oligonucleotides** |  |  |
| Primer for *CPT1ab* knockout upstream primer,  5'-TAATACGACTCACTATAGGGCCTCGTGG  CACAGATGCGTTTTAGAG-3'  Primer for *CPT1ab* knockout downstream primer,  5'-AGCACCGACTCGGTGCCACT-3'  Primer for siACLY forward primer,  5’-CAGGGUGAAAUCGAUUAAUTT-3’  Primer for siACLY reverse primer,  5’-AUUAAUCGAUUUCACCCUGTT-3’  Primer for siRaptor forward primer,  5’-GCCUCGACCUACUGUUAAUTT-3’  Primer for siRaptor reverse primer,  5’-AUUAACAGUAGGUCGAGGCTT-3’  Primer for sip300 forward primer,  5’-GCCUCAUCCAGACAGAUAATT-3’  Primer for sip300 reverse primer,  5’-UUAUCUGUCUGGAUGAGGCTT-3’  Primer for siGCN5 forward primer,  5’-GGCAUACAAAGUCGAUUAUTT-3’  Primer for siGCN5 reverse primer,  5’-AUAAUCGACUUUGUAUGCCTT-3’ | This paper  This paper  This paper  This paper  This paper  This paper  This paper  This paper  This paper  This paper | N/A  N/A  N/A  N/A  N/A  N/A    N/A  N/A  N/A  N/A |
