# Supplementary material for: Inhibition of mitochondrial fatty acid β-oxidation activates mTORC1 pathway and protein synthesis via Gcn5-dependent acetylation of Raptor in zebrafish
Source: J Biol Chem. 2023 Sep 3;299(10):105220. doi: 10.1016/j.jbc.2023.105220 (PMC10540046; doi:10.1016/j.jbc.2023.105220)
Supplement: Supplemental Table S3 [file mmc3.docx]

**Table S3**. Primer sequences accession numbers for real-time PCR analysis.

| Gene | Forward primer (5' to 3') | Reverse primer (5' to 3') | Gene ID |
| --- | --- | --- | --- |
| *mpc1*  *pk*（liver）  *pk*（muscle）  *pdh*  *acly*  *raptor*  *p300a*  *p300b*  *cbpa*  *cbpb*  *gcn5*  *pcaf*  *kat5a*  *kat5b*  *kat6a*  *kat6b*  *kat7a*  *kat7b*  *hdac4*  *hdac7*  *hdac9*  *β-actin*  *ef1a* | GCGCAAAGCTGTGGATCATC  ATCACTGCCCGCAACACCA  TGAACATCGCTCGCATGAACTT  AAGCGTGTGCCGTGGGTATTG  AGACCTGATCTCCAGCCTCACATC  AAACCCAGGAGTGCCATCAG  CGAGCAGATATGGCAGACGA  GACAAGAAGCCGGAGGTCAA  CTCCAACGCAAGGGATGCTA  CGGGGGCATGAATGTGAATG  AATGACAGCTTGCCACGGTA  CAGGCGGACAGATTCCAACA  GAGGCAAAGACCCCCATGAA  TCCACCGTCTATTTGCCACC  TGATGACCAACCTCATGCCC  CCCCCAATCAACAAGAAAACCA  CTCGTAGTCAACAGCAGGGG  CGGAAGGGCCATAAATCTCCA  GAGAGCGAGACGGGGAATGTG  GGGGTGGACAATGACACCAT  AAAATCAGCCAGTGTGGGGT  GTCATCACCATCGGCAAT  CCCCTGGACACAGAGACTTCATC | TGGCCATCTTCTTCTCCATGT  TCATTCCTGCTTTCACCATCTCC  TCAAAGCTGGCACAAGCTTCA  GTGCCTCCTCTGGTGAGTGTGT  ATGCCACTGTCGAATGCCTTACTG  AGCTAAACCTCGTGTGGTGG  TACAATTCAGCGAGCGGAGG  AGTAGTCCAGGTTAGTTTTGTTACT  CCAAGTGCATTCAGGGGGAG  AGCGTTCAGGGTCAGGTTAG  TGAGGATGAGGGTGCGTTTC  ATGTCACCAACCACACGAGG  CTCTTGAAGGAGCCGAAGCA  TTGCATTTAGTCAGGTGCCTCT  TAGGGCGGGTTACTGTGGTA  AGGAGAACGCTAGACGGGG  GCGGTTTTCTCCTCCTACCC  GTCCCGTCTGAACTACTGCC  GGGTTACAACACGCGCACAG  TCGCCACCGAGTTGAAGAAA  GCTCGTTTGCGGGATGTTAT  CGTGGATACCGCAAGATT  ATACCAGCCTCAAACTCACCGAC | 436671  114551  335817  406702  436922  560455  559273  565612  566841  567111  555517  563942  436638  503731  266966  568932  767644  323181  568877  798603  393789  57934  30516 |

Mitochondrial pyruvate carrier 1 (*mpc1*); Pyruvate kinase (*pk*); pyruvate dehydrogenase (*pdh*); ATP citrate lyase (*acly*); *raptor*: regulatory-associated protein of mTORC1; Acetyltransferase family: *p300a*, *p300b*, *cbpa*, *cbpb*, *gcn5*, *pcaf*, *kat5a*, *kat5b*, *kat6a*, *kat6b*, *kat7a*, *kat7b*; Deacetylase family: *hdac4*, *hdac7*, *hdac9*; Reference gene: ongationfactor1-alpha (*ef1α*); *β-actin*.
