# Supplementary material for: Inhibition of mitochondrial fatty acid β-oxidation activates mTORC1 pathway and protein synthesis via Gcn5-dependent acetylation of Raptor in zebrafish
Source: J Biol Chem. 2023 Sep 3;299(10):105220. doi: 10.1016/j.jbc.2023.105220 (PMC10540046; doi:10.1016/j.jbc.2023.105220)
Supplement: Supplemental Figure Legend [file mmc4.docx]

**Figure S1.**

(A-G) The effects of mitochondrial FAO inhibition by mildronate (MD) on liver carnitine content (A, n=5), muscle carnitine content (B, n=5), liver TG content (C, n=5), muscle TG content (D, n=5), liver glycogen content (E, n=5), muscle glycogen content (F, n=5), and weight gain (WG) (G, n=9) in zebrafish. WG = 100 × (final mean body weight – initial mean body weight)/initial mean body weight.

(H) The protein expression level of Cpt1a in *cpt1ab* -/- zebrafish muscle tissue (CRISPR/Cas9 editing). N=3.

(I and J) The carcass (C, n=7) and carcass protein content (D, n=5) of WT and *cpt1ab* -/- zebrafish.

(K) The effect of *cpt1ab* -/- on mTORC1 signaling pathway (p-S6k**^Thr389^** and p-S6**^Ser235/236^**) in zebrafish muscle. N=3.

(L and M) mTORC1 activity (p-mTOR**^Ser2448^**, p-S6**^Ser235/236^**) (L, n=3) and relative growth rate (M, n=3) in CN and MD treated ZFL cells.

(N and O) mTORC1 activity (p-mTOR**^Ser2448^**, p-S6**^Ser235/236^**) (N, n=3) and relative growth rate (O, n=7) in CN and MD treated ZFM cells. (P and Q) mTORC1 activity (p-mTOR**^Ser2448^** and p-S6k**^Thr389^**) (Q, n=3) and relative growth rate (R, n=6) in CN and Etomoxir treated ZFL cells. Note: The concentration and time of Etomoxir treated cells were 1uM and 24h (the same below).

Data represent mean ± SD. ^∗^*p* < 0.05, ^∗∗^*p* < 0.01.

**Figure S2.**

(A) Relative *mRNA* expression of *pk* and *pdh* for glucose catabolism in CN- and MD-fed zebrafish liver and muscle tissues. N=6.

(B-D) Supernatant pyruvate content (B, n=3), intracellular acetyl-CoA level (C, n=3) and global protein lysine acetylation (D, n=3) in CN and MD treated ZFM cells.

(E and F) Acetyl-CoA level (E, n=3) and global protein lysine acetylation level (F, n=3) in CN- and MD-fed zebrafish liver and muscle tissues.

(G) The acetyl-CoA level in muscle tissue from WT and *cpt1ab* -/- zebrafish. N=3.

(H and I) The global protein lysine acetylation (H) and relative protein quantification level (I) in WT and *cpt1ab* -/- zebrafish muscle tissue. N=3.

(J-L) Supernatant pyruvate content (J, n=3), acetyl-CoA level (K, n=3) and global protein lysine acetylation level (L, n=3) in CN and Etomoxir treated ZFL cells.

Data represent mean ± SD. ^∗^*p* < 0.05, ^∗∗^*p* < 0.01.

**Figure S3.**

(A) The Raptor acetylation level in CN and MD treated ZFM cells. N=3.

(B and C) Western blotting and relative quantification of Raptor acetylation level in CN- and MD-fed zebrafish liver and muscle tissues. N=3.

(D) The Raptor acetylation level in WT and *cpt1ab* -/- zebrafish muscle tissue. N=3.

(E) The Raptor acetylation level in CN and Etomoxir treated ZFM cells. N=3.

Data represent mean ± SD. ^∗^*p* < 0.05, ^∗∗^*p* < 0.01.

**Figure S4.**

(A-C) The effects of Gcn5/Pcaf inhibitor (CPTH6, 1uM) treatment on global proteins lysine acetylation (A), protein quantification of acetyltransferase (P300, Gcn5 and Pcaf) (B), and Raptor acetylation (C) and N=3.

(D) The effects of CPTH6 on mTORC1 activity (p-mTOR**^Ser2448^**, p-S6**^Ser235/236^** and p-4ebp**^Thr37/46^**) in ZFL cells. N = 3.

(E) Relative mRNA levels of *gcn5* in negative control (NC) and Raptor siRNA treated ZFL cells. N=5.

Data represent mean ± SD. ^∗^*p* < 0.05, ^∗∗^*p* < 0.01.

**Figure S5.**

(A) The effect of *p300* siRNA treatment on Raptor acetylation in ZFL cells. N=3.

(B) The effect of P300 inhibitor (C646, 1uM) treatment on Raptor acetylation in ZFL cells. N=3.

(C) Western blotting and relative protein quantification of P300, Gcn5, Pcaf, p-S6**^Ser235/236^** and S6 in C646-treated ZFL cells. N = 3.

(D) The effect of MYST inhibitor (WM1119, 10uM) treatment on Raptor acetylation in ZFL cells. N=3.

(E) Western blotting and relative protein quantification of P300, Gcn5 and Pcaf in CN- and MYST-treated ZFL cells. N = 3.

(F) The effects of WM1119 on mTORC1 activity (p-S6**^Ser235/236^** and p-4ebp**^Thr37/46^**) in ZFL cells. N = 3.

Data represent mean ± SD. ^∗^*p* < 0.05, ^∗∗^*p* < 0.01.

**Figure S6.**

(A) Relative protein quantification of p-S6**^Ser235/236^** and S6 in CN, C646 and C646 with CPTH6 treated ZFL cells. N = 3.

(B) Relative protein quantification of p-S6**^Ser235/236^** and S6 in CN, WM1119 and WM1119 with CPTH6 treated ZFL cells. N = 3.

Data represent mean ± SD. ^∗^*p* < 0.05, ^∗∗^*p* < 0.01.
